# Humoral Immunogenicity to SARS-CoV-2 Vaccination in Liver Transplant Recipients: A Systematic Review and Meta-Analysis

**DOI:** 10.7150/ijbs.77030

**Published:** 2022-09-21

**Authors:** Jeong-Ju Yoo, Dong Keon Yon, Seung Won Lee, Jae Il Shin, Beom Kyung Kim

**Affiliations:** 1 Department of Internal Medicine, Soonchunhyang University Bucheon Hospital, Soonchunhyaung University College of Medicine, Bucheon, Republic of Korea.; 2 Department of Pediatrics, Kyung Hee University Hospital, Kyung Hee University College of Medicine, Seoul, Republic of Korea.; 3 Department of Data Science, Sejong University College of Software Convergence, Seoul, Republic of Korea.; 4 Sungkyunkwan University School of Medicine, Suwon, Republic of Korea.; 5 Department of Pediatrics, Yonsei University College of Medicine, Seoul, Republic of Korea.; 6 Department of Internal Medicine, Yonsei University College of Medicine, Seoul, Republic of Korea.; 7 Institute of Gastroenterology, Yonsei University College of Medicine, Seoul, Republic of Korea.; 8 Yonsei Liver Center, Severance Hospital, Yonsei University Health System, Seoul, Republic of Korea.

**Keywords:** Meta-analysis, liver transplant, vaccine, SARS-CoV-2, immunogenicity

## Abstract

Solid organ transplant recipients generally show reduced immunogenicity to various vaccines. We aimed to assess the immunogenicity of the immune response among orthotopic liver transplant (OLT) recipients after the severe acute respiratory syndrome coronavirus 2 (SARS-CoV-2) vaccination. A systematic search was performed to evaluate immunogenicity or adverse events reported after SARS-CoV-2 vaccination. The pooled analysis of 20 studies showed a humoral immune response rate of 0.70 (95% confidence interval [CI], 0.63-0.77) after SARS-CoV-2 vaccination among OLT recipients. The immunogenicity among OLT recipients was significantly lower compared to the overall population and healthy controls, with odds ratios (ORs) of 0.80 and 0.69. However, it was significantly higher than that of patients receiving other organ transplants, especially kidneys, with an OR of 1.50. Male sex, old age, chronic kidney disease, obesity, and multiple or high immunosuppressant doses significantly increased the risk of unresponsiveness in patients with OLT. The overall incidence of any adverse event after vaccination was 0.68 (95% CI, 0.55-0.81), similar to that of control. OLT recipients had an overall humoral immune response rate of 70% after SARS-CoV-2 vaccination, which is lower than that of healthy controls but favourable compared to those of other solid organ transplant recipients.

## Introduction

Coronavirus disease 2019 (COVID-19), which is caused by severe acute respiratory syndrome coronavirus 2 (SARS-CoV-2), has led to more than 6 million deaths, dramatically increasing the burden on healthcare systems worldwide 1-5. Since vaccination against SARS-CoV-2 is the most appropriate way to achieve herd immunity, more than 5 billion people globally have received at least one dose as of April 2022. Considering that patients who had organ (or liver) transplant have worse COVID-19 outcomes than general population, vaccination against SARS-CoV-2 is generally recommended for this population 6-10.

In contrast, solid organ transplant recipients generally show reduced immunogenicity to a number of vaccines. For example, extensive studies on influenza vaccines have shown that antibody- and cell-mediated immune responses are lower in solid organ transplant recipients than in the general population 11,12. This is primarily due to inhibited lymphocyte activation, interaction with antigen-presenting cells, and decreased B-cell memory responses led by the lifelong administration of immunosuppressive agents 13. Nevertheless, vaccination against influenza has been associated with a reduction in influenza-associated complications in patients receiving solid organ transplants. Likewise, although the overall response rate of the first dose of mRNA vaccine against SARS-CoV-2 among solid organ transplant recipients was disappointingly low, it seems prudent to vaccinate immunocompromised patients since the benefits outweigh the risks 14. Only 17% of these vaccinated individuals achieved detectable antibodies to the SARS-CoV-2 spike protein, although the response rate might vary depending on the type of transplanted organ and/or immunosuppressive agents 13. However, pooled analyses of serial vaccine pharmacodynamics, effectiveness, and response duration in terms of humoral or cellular immunity against SARS-CoV-2 specified for populations receiving orthotopic liver transplants (OLT) are currently scarce.

Therefore, herein we aimed to explore the trends of humoral immune response immunogenicity among patients receiving OLT after completion of the SARS-CoV-2 vaccine series and assess the identified risk factors for vaccine nonresponse.

## Materials and Methods

The protocol for this review was registered with PROSPERO (International Prospective Register of Systematic Reviews, CRD42022324652) in advance. This systematic review and meta-analysis were performed according to the Meta-analysis of Observational Studies in Epidemiology (MOOSE) checklist and Preferred Reporting Items for Systematic Reviews and Meta-Analyses (PRISMA) guidelines.

### Inclusion criteria, exclusion criteria, and study outcomes

Studies were included if they were randomised controlled trials, cross-sectional studies, or cohort studies, including those of prospective and retrospective designs that reported on the immunogenicity of COVID-19 vaccination in adult OLT recipients (>19 years old) of deceased or living donor liver transplants. For the COVID-19 vaccine, both mRNA and viral vector vaccines were investigated. The exclusion criteria were as follows: i) case reports, ii) case series of fewer than five patients, and iii) reviews. The primary outcome of interest was the proportion of liver transplant recipients with a serological antibody response to a full or partial COVID-19 vaccination dose.

### Search strategy

We searched for synonymous terms and used them to develop the search strategy. The keywords used in the Patient/Problem, Intervention, Comparison, and Outcome (PICO) model are shown in the Supplementary material and method section. We searched the PubMed (Medline), EMBASE, and Cochrane Library databases using terms Medical Subject Headings (MeSH) terms to identify studies published in English between 1 January 2019 and 31 March 2022. The search strategies and results of each database search are shown in the Supplementary material and method section. The search terms included liver transplantation-related index words and COVID-19 vaccine-related index words.

### Study selection and data extraction

Two authors independently screened the titles and abstracts. Two reviewers (BKK and JJY) independently screened the full-text articles for study relevance. Any discrepancy between the two reviewers was resolved by JIS or JYK after discussion. The two researchers also independently performed the risk of bias assessment and extracted the study data, including the characteristics and results, and recorded them in a standard form.

### Methodological quality and risk of bias assessment

We used the Risk of Bias Assessment tool for Nonrandomised Studies (RoBANS) 15 to assess the risk of bias; the overall results are shown in the Supplementary material risk of bias section. Any discrepancy was resolved by two authors (BKK and JJY) after discussion. Publication bias was assessed using funnel plots (Figure S1, S2).

### Statistical analysis

The pooled prevalence was derived using a random-effects model. Characteristics were compared between the OLT and control groups using a random-effects model as the risk ratio for continuous variables and the Freeman-Tukey variant of the arcsine square root transformed proportion for binary variables. 16 The risk factors were recorded as odds ratios (ORs) with 95% confidence intervals. We evaluated inter-study heterogeneity using the I^2^ metric of inconsistency and the P value of the Cochran Q test. I^2^, the ratio of inter-study variance to the sum of intra-study and inter-study variance, ranges from 0% to 100%. To explain inter-study heterogeneity, a meta-regression was conducted to examine the influence of other factors on clinical outcomes. Statistical analyses were performed using RevMan 5 (Cochrane Library) or the meta package in R (version 4.1.0; R Foundation for Statistical Computing, Vienna, Austria).

## Results

### Characteristics of included studies

Based on the title and abstract screening, we identified 26 potentially relevant studies. Among them, six were excluded for the following reasons: wrong patient population (n=2), overlapping populations (n=2), case reports (n=2), and reviews (n=1). As a result, 20 studies were included in the meta-analysis (Figure S3). Information regarding the enrolled patients is presented in **Table 1**.

As shown in Table 1, all 20 studies were conducted in various countries, mostly within Europe (n=11), followed by North America (n=4), the Middle East (n=3), and East Asia (n=2). In most studies, patients with a prior history of COVID-19 infection were excluded from the analysis. The Pfizer vaccine alone was the most common vaccine used in the study (n=9), followed by the mRNA vaccine (Pfizer or Moderna, n=5), mRNA vaccine or vector vaccine (n=4), and Moderna vaccine alone (n=2). Anti-S immunoglobulin G (IgG) was most commonly used as an evaluation tool for immunogenicity, and anti-RBD IgG or anti-nucleocapsid IgG was used.

### Immunogenicity rates of COVID-19 vaccination among OLT recipients

**Figure 1:**
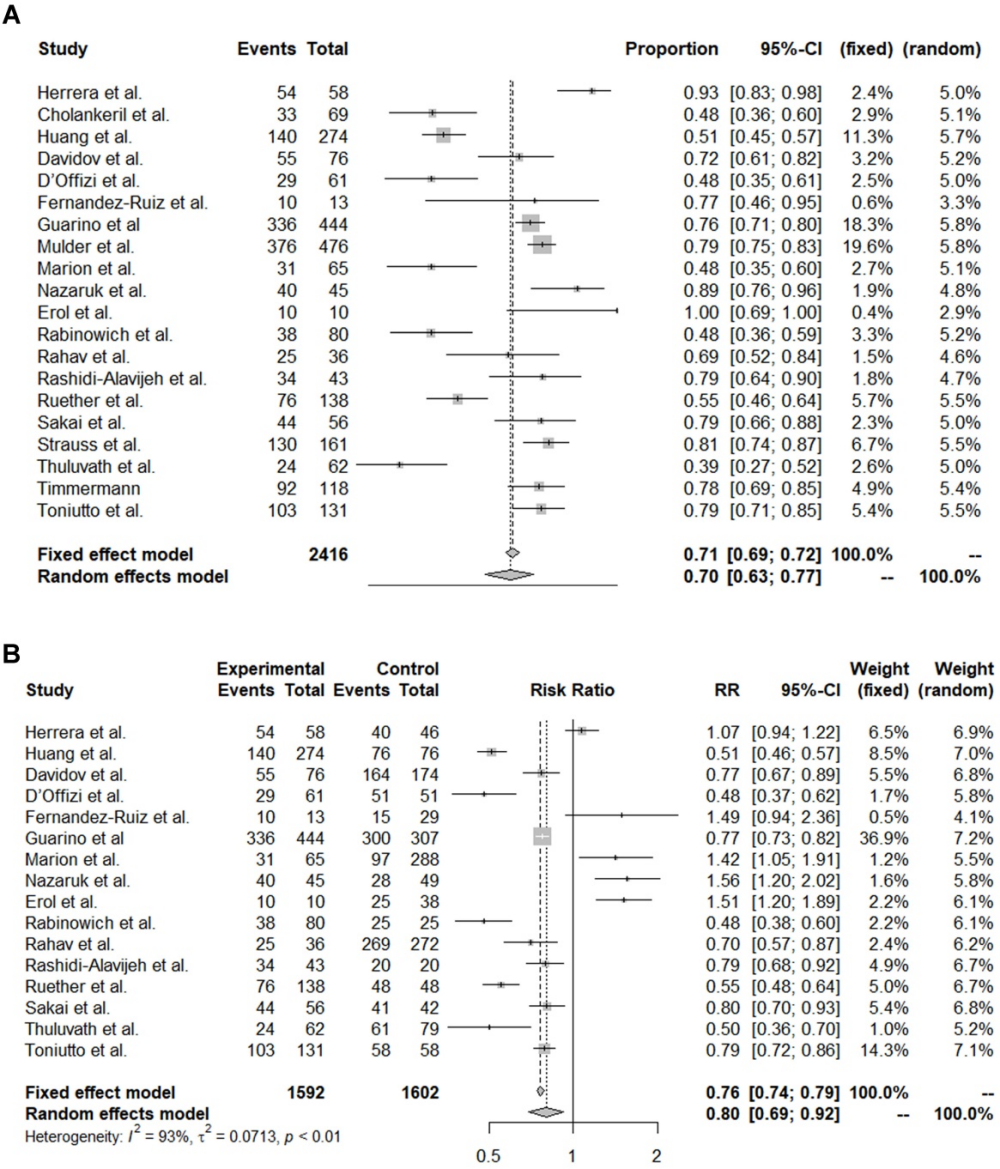
** Forest plots of immunogenicity rates. (A)** Pooled immunogenicity rate. **(B)** Comparison of liver transplantation recipient and control groups.

The pooled immunogenicity rate of OLT recipients against the COVID-19 vaccine was 0.70 (95% CI, 0.63-0.77) (**Table 2, Figure 1A**). A stratified analysis of the countries of the study subjects revealed that the immunogenicity of European (event rate, 0.74) and Asian (event rate, 0.56) patients was higher than that of American (event rate, 0.56) and the Middle Eastern (event rate, 0.63) patients. By vaccine type, the immunogenicity rate of Moderna (event rate, 0.88) and Pfizer alone (event rate, 0.71) was higher than that of the mRNA mixed group (event rate, 0.66) and the mRNA + vector vaccine group (event rate, 0.64). On the other hand, there was no significant difference in the immunogenicity rate by study design, year of publication, or diagnostic tool.

Next, we analysed how the immunogenicity of the OLT patient group differed from that of the control groups (Table S1, Figure 1B). Among the 20 papers, 16 reported differences in immunogenicity between OLT and control groups. The immunogenicity of the OLT recipients was significantly lower than that of the overall control group, with an OR of 0.80 (95% CI, 0.69-0.92). In particular, the immunogenicity of OLT recipients was significantly lower than that of healthy controls (pooled OR from 10 studies, 0.69; 95% CI, 0.63-0.77) or liver cirrhosis patients (pooled OR from 2 studies, 0.54; 95% CI, 0.47-0.62). On the other hand, the immunogenicity rate of OLT recipients was significantly higher than that of recipients of other organ transplants, such as kidneys (pooled OR from 6 studies, 1.50; 95% CI, 1.35-1.67) or hearts (pooled OR from 3 studies, 1.44; 95% CI, 0.89-2.32).

Finally, we performed a meta-regression analysis because of the high inter-study heterogeneity (Table S2). The analysis showed no association of the pooled adjusted OR with age, sex, body mass index, comorbidities, and immunosuppressant type or number.

### Risk factors for the unresponsiveness to vaccination among OLT recipients

We identified all risk factors described in the studies. In our meta-analysis, a total of 10 risk factors were accessible for calculation: sex, age, chronic kidney disease (CKD), obesity, multiple immunosuppressant use, high steroid dose, mycophenolate mofetil, tacrolimus, time since OLT, and vaccination in 1^st^ year after transplantation (Table S3). All risk factors but high-dose tacrolimus use were significantly associated with vaccine unresponsiveness among OLT recipients. In particular, CKD (OR, 27.56; 95% CI, 10.06-87.54), vaccination in 1^st^ year after transplantation (OR, 18.53; 95% CI, 7.67-44.79), and the use of multiple immunosuppressants (OR, 10.40; 95% CI, 6.12-17.68) significantly increased the risk of unresponsiveness.

### Adverse events after COVID-19 vaccination among OLT recipients

**Figure 2:**
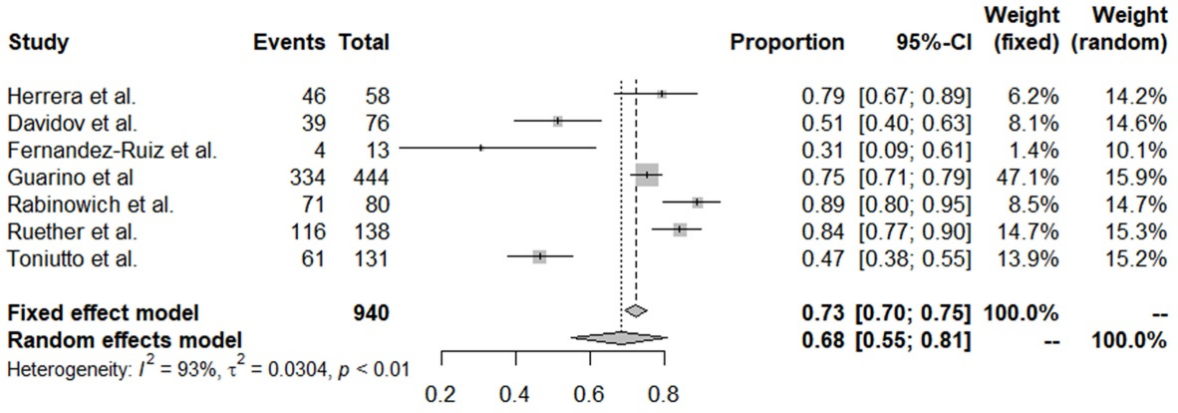
Forest plots of all adverse events reported after coronavirus disease 2019 vaccine administration.

Of the 20 studies, 7 reported adverse events after vaccination. The overall incidence of adverse events was 0.68 (95% CI, 0.55-0.81) (Table 3, Figure 2). The incidence of these adverse events did not differ significantly by country, vaccine type, or publication year. Serious adverse events (SAEs) were reported in 9 papers; 1 patient for Bell's palsy 17 and 6 patients for joint pain/fever, fatigue/headache/muscle pain requiring hospitalization 11, 18 were reported in 2 papers, but there was no occurrence of SAE in the remaining 7 papers. Also, no deaths have been reported in these vaccinated patients and there were no studies reporting the rate of COVID-19 infection after vaccination.

## Discussion

In the present systematic review and meta-analysis, we observed that OLT recipients had a 70% (95% CI, 0.68-0.77) overall humoral immune response after vaccination against SARS-CoV-2, which was significantly lower than that of the controls, with a risk ratio (RR, 0.80; 95% CI, 0.69-0.92). In particular, compared to that of healthy controls, the OR of 0.69 (95% CI, 0.63-0.77) of the vaccine response among OLT recipients was relatively lower. However, compared to patients receiving transplants of other solid organs such as kidneys or hearts, OLT recipients showed a trend toward relatively favourable responses with an OR of 1.50 (95% CI, 1.35-1.67) and 1.44 (95% CI, 0.89-2.32), respectively. There are several possible explanations for this finding. First, it might be related to the fact that most thoracic or kidney transplant patients receive induction therapy 19,20, whereas only less than 30% of OLT recipients receive induction therapy 21. In a similar context, we found that OLT patients receiving calcineurin inhibitor-based immunosuppressive regimens were more likely to be responders than those receiving a mycophenolate mofetil-based regimen 22. Lastly, liver is the major immune-modulating organ, thus, restoring liver function after OLT could be more beneficial in achieving immune response after the vaccination 23.

Notably, among various risk factors, the presence of CKD showed the highest impact on non-response to vaccine, with a pooled OR of 27.56 (95% CI 10.06-87.54). Even in patients with CKD who are not receiving immunosuppressive agents, impaired adaptive immunity is often observed. Antibody production by B lymphocytes decreases with the dysfunction of antigen-presenting cells and memory T cell apoptosis under uremic conditions 24. Furthermore, both insufficient erythropoietin (EPO) and vitamin D can, in part, contribute to the dysregulation of immunomodulation 25. Considering that OLT recipients are subject to CKD development during their life owing to nephrotoxic calcineurin inhibitors as well as peri-operative medical condition 26-29, the presence of CKD itself among OLT recipients might have an additional impact compared with other risk factors. However, further studies are required to determine whether therapeutic intervention with EPO and vitamin D supplementation in OLT patients with CKD might positively affect the efficacy of the SARS-CoV-2 vaccine. In addition, the depth of immunosuppression, which was also closely associated with a higher non-response when the vaccine was received within the 1^st^ year after transplantation, had a strong impact on the humoral response. In our meta-regression analysis, the use of one immunosuppressive agent increased the humoral immune response compared with the use of two or more immunosuppressive agents (p=0.002). In addition, the presence of CKD negatively impacted the immune response with marginal significance (p=0.052). Nevertheless, solid organ transplantation experts 30,31 recommend the maintenance of immunosuppressive agents, including mycophenolate mofetil) when recipients of solid organs receive the SARS-CoV-2 vaccination owing to the potential concern of graft rejection. Further clinical trials are needed to elucidate this issue.

In terms of adverse events, about 68% of OLT recipients experienced any kinds of adverse events. Although the currently approved SARS-CoV-2 vaccines include novel mRNA types, there are no theoretical concerns that they should not be safe for immunosuppressed individuals, as they contain no live viruses capable of replication within the vaccinated host. In the first studies assessing the safety of SARS-CoV-2 mRNA vaccines among patients who received solid organ transplants, the type and rate of adverse events have been similar to those of non-immunosuppressed controls (70-85% of pain at the injection site and 15-20% of fatigue) 32-34.

In general, more than 20% of patients with cirrhosis show unresponsiveness to the SARS-CoV-2 vaccination 35 because they are also considered immunocompromised. Hepatic fibrosis impairs the synthesis of innate immunity proteins and pattern recognition receptors, and the absolute counts and functions of B and T lymphocytes are affected by diverse mechanisms, including co-stimulation marker downregulation, memory cell loss, and T cell exhaustion 35,36. Nevertheless, notably, OLT patients showed significantly lower immunogenicity than patients with cirrhosis (OR, 0.54; 95% CI, 0.47-0.62). This indicates that the use of immunosuppressive agents in the presence of other associated comorbidities including renal insufficiency (either primary or secondary to underlying liver disease) 37-41, should offset the benefit of OLT, that is, restored hepatic functional reserve and normalised portal pressure in achieving a humoral response to the SARS-CoV-2 vaccination.

This study had several limitations. First, we could not assess the cellular immune response by T cells among the OLT recipients. There were only 4 articles reporting the cellular immunogenicity in these patients' group. However, it was impossible to derive an integrated result appropriately, since the evaluation tool for cellular response was heterogeneous among studies. Although a serological response to a virus-specific antibody is generally the major endpoint in the evaluation of vaccine efficacy 42, coordinated and lasting CD4+ and CD8+ T cell responses with the proper specificity, phenotype, and function are also likely critical components of antiviral immunity against SARS-CoV-2 43 since circulating antibodies to SARS-CoV-2 may be short-lived or of low magnitude and/or potency 44,45. Further studies measuring the secretion of interferon-gamma by peripheral blood lymphocytes upon SARS-CoV-2 glycoprotein stimulation are required to resolve this issue. In a similar context, further studies are required to determine whether booster doses of vaccines based on mRNA or viral vectors can affect the overall humoral and cellular immune responses among OLT recipients. Similarly, whether there is a correlation between diminishing antibody levels after vaccination and SARS-CoV-2 infection susceptibility or severity should be assessed through further research. In addition, although the rate of achieving immune response could be the only available indicator at present, data regarding other clinical outcomes such as rate of SARS-CoV-2 infection after vaccination, complication or mortality have been still insufficient so far. Further long-term follow-up should be required to address this issue.

In conclusion, because OLT recipients are at risk of developing a higher rate of COVID-19-related complications, they are at priority for receiving immunisations against SARS-CoV-2. The humoral response to vaccines against SARS-CoV-2 among OLT recipients was relatively lower than that of healthy controls, with a similar level of adverse events compared to the historical controls. However, OLT recipients had a more favourable response than patients who received kidney or heart transplants. Interventions to achieve higher response rates, such as booster vaccinations, higher vaccine dosages, and intradermal administration, require assessment in well-designed clinical trials.

## Supplementary Material

Supplementary materials and methods, figures and tables.

## Figures and Tables

**Table 1:** Demographics and characteristics of studies included in the systematic review and meta-analysis

Study	Country	Study design	Inclusion	Exclusion	Vaccine type	Dose	Response evaluation	Objective indicator of humoral immunogenicity	Antibodies	LT duration (yrs)	Age (median)	Male (%)
Herrera (2021) 46	Spain	Prospective	heart and liver transplant recipients	prior COVID	Moderna	2	4 weeks after 2^nd^ vaccine	Presence of anti-S IgG spike	Anti-S IgG	4.6	61.5	73
Cholankeril (2021) 47	USA	Prospective	LT recipients	prior COVID	Pfizer	2	30 to 75 days after 2^nd^ vaccine	Semi-quantitative anti-S IgG value > 1	Anti-S IgGAnti-nucleocapsid IgG	3.3	63	70
Huang (2002) 48	USA	Retrospective	adult organ transplantation	prior COVID	mRNA vaccine (Pfizer or Moderna)	2	day 30-90 after 2^nd^ vaccine	anti-S IgG titer > 1:50	Anti-S IgGAnti-nucleocapsid IgG	3.2	62	60.7
Davidov (2022) 17	Israel	Prospective	LT recipients	prior COVID	Pfizer	2	36 days after 2^nd^ vaccine	anti-RBD IgG titers > 1.1 sample-to-cutoff ratio /neutralizing antibodies	Anti-RBD IgGNeutralizing antibodies	6	64	56.6
D'Offizi (2021) 49	Italy	Retrospective	LT recipients	prior COVID	mRNA vaccine (Pfizer or Moderna)	2	2 weeks after 2^nd^ vaccine	anti-S IgG > 7.2 BAU/mL	Anti-S IgG	6	59	70
Fernández-Ruiz (2021) 50	Spain	Prospective	KT or LT recipients	prior COVID	Moderna	2	2 weeks after 2^nd^ vaccine	Presence of anti-S IgG spike	Anti-S IgG			
Guarino (2022) 51	Italy	Prospective	LT recipients	prior COVID	Pfizer	2	1 month and 3 months after 2^nd^ vaccine	anti-S IgG >25 AU/mL	Anti-S IgG	14.08	64.8	75.4
Mulder (2002) 52	Netherlands	Retrospective	LT recipients	prior COVID	mRNA vaccine or ChAdOx1 nCoV-19	2	4 weeks after 2^nd^ vaccine	Presence of anti-S IgG spike	Anti-S IgG	5.5	59	60
Marion (2022) 22	France	Retrospective	sold organ transplantation		mRNA vaccine (Pfizer and Moderna)	2	4 weeks after 2^nd^ vaccine	Presence of anti-S IgG spike	Anti-S IgG			
Nazaruk (2021) 53	Poland	Retrospective	KT or LT recipients	prior COVID	Pfizer	2	4-8 weeks after the 2^nd^ vaccine	anti-S IgG > 50 AU/mL /neutralizing antibodies	Anti-S IgGNeutralizing antibodies	14.8	58.4	80
Erol (2021) 54	Turkey	Prospective	KT or LT recipients	prior COVID	Sinovac or Pfizer	2	4-6 weeks after the 2^nd^ vaccine	anti-S IgG > 50 AU/mL	Anti-S IgG			
Rabinowich (2021) 32	Israel	Prospective	LT recipients		Pfizer	2	10-20 days after the 2^nd^ vaccine	anti-S IgG > 15 AU/mL /anti-nucleocapsid IgG	Anti-S IgGAnti-nucleocapsid IgG	5	60.1	30
Rahav (2021) 55	Israel	Prospective	immunocompromised	prior COVID	Pfizer	2	2-4 weeks after the 2^nd^ vaccine	anti-RBD IgG titers > 1.1 /neutralizing antibodies	Anti-RBD IgGNeutralizing antibodies		68	52.8
Rashidi-Alavijeh (2021) 56	Germany	Prospective	LT recipients		Pfizer	2	15 days after the 2^nd^ vaccine	anti-S IgG > 13 AU/mL	Anti-S IgG	8	57	60.5
Ruether (2022) 18	Germany	Prospective	LT recipients or liver cirrhosis		mRNA vaccine or vector-based vaccine(AZD1222; AstraZeneca)	2	4 weeks after the 2^nd^ vaccine	anti-S IgG > 33.8 BAU/mL /anti-RBD IgG	Anti-S IgGAnti-RBD IgG	17	55	57.2
Sakai (2022) 57	Japan	Retrospective	LC or LT recipients	prior COVID	Pfizer	2	2 weeks after the 2^nd^ vaccine	anti-RBD IgG titers > 1.0 AU/mL	Anti-RBD IgG	15.5	65	76.8
Strauss (2021) 58	USA	Retrospective	LT recipients		mRNA vaccine (Pfizer or Moderna)	2	4 weeks after the 2^nd^ vaccine	anti-S1 IgG > 1.1 AU/mL /anti-RBD IgG > 0.8 U/mL	Anti-S1 IgGAnti-RBD IgG	6.9	64	43
Thuluvath (2021) 59	USA	Prospective	LT recipients and those with chronic liver disease (CLD) with andwithout cirrhosis	prior COVID	mRNAvaccines or after the single dose of Johnson & Johnson vaccine	2	4 weeks after the 2^nd^ vaccine	anti-S IgG > 0.4 U/mL	Anti-S IgG		65.7	66
Timmermann (2021) 60	Germany	Retrospective	LT recipients		mRNAvaccines or after the single dose of Johnson & Johnson vaccine	2	3 weeks after the 2^nd^ vaccine	Presence of anti-S IgG spike/anti-nucleocapsid IgG	Anti-S IgGAnti-nucleocapsid IgG	14.4	66.1	63.6
Toniutto (2022) 61	Italy	Retrospective	LT recipients	prior COVID	Pfizer	2	1, 4, 6 month after the 2^nd^ vaccine	anti-RBD IgG > 0.8 U/mL /anti-nucleocapsid IgG > 0.8 > 10 kAU/L	Anti-RBD IgGAnti-nucleocapsid IgG	7.8	57.9	70.2

Abbreviations: LT, liver transplantation; KT, kidney transplantation; RBD, receptor binding protein.

**Table 2:** Summary of the immunogenicity rates of COVID-vaccination in patients with liver transplantation recipients

Subgroup/Subset	No. of studies	No. of patients responder/total	Pooled event rate (M-H, Random)	95% CI	*I* ^2^	P for heterogeneity
**Overall**						
Immunogenicity rate, overall	20	1,680/2,416	0.70	0.63 to 0.77	91%	<0.01
**Country**						
Europe	11	1,181/1,592	0.74	0.66 to 0.81	89%	<0.01
America	4	327/566	0.56	0.36 to 0.74	95%	<0.01
Middle east	3	118/192	0.63	0.46 to 0.79	82%	<0.01
East	2	54/66	0.90	0.62 to 1.00	75%	0.05
**Types of vaccine**						
Moderna only	2	64/71	0.88	0.69 to 1.00	61%	0.11
Pfizer only	9	708/980	0.71	0.63 to 0.79	85%	<0.01
mRNA vaccine (Moderna, Pfizer or Sinovac)	5	350/571	0.66	0.47 to 0.82	94%	<0.01
mRNA vaccines + vector vaccines	4	568/794	0.64	0.46 to 0.80	95%	<0.01
**Study design**						
Prospective	11	608/1029	0.69	0.58 to 0.79	91%	<0.01
Retrospective	9	985/1387	0.71	0.61 to 0.80	93%	<0.01
**Publish year**						
2021	12	519/756	0.72	0.60 to 0.83	91%	<0.01
2022	8	1161/1660	0.68	0.58 to 0.77	93%	<0.01
**Diagnosis tool**						
Anti-Spike immunoglobulin	16	1453/2117	0.69	0.60 to 0.77	93%	<0.01
Anti-RBD immunoglobulin	4	227/299	0.76	0.71 to 0.81	0%	0.55

* CI: confidence interval; M-H: Mantel-Haenszel; No.: number; RR: risk ratio.

**Table 3:** Adverse event of COVID-vaccination in patients with liver transplantation recipients

Subgroup/Subset	No. of studies	No. of patients AE/total	Pooled event rate (M-H, Random)	95% CI	*I* ^2^	P for heterogeneity
**Overall**						
Adverse events, overall	7	671/940	0.68	0.55 to 0.81	93%	<0.01
**Country**						
Europe	5	561/784	0.67	0.51 to 0.81	93%	<0.01
Middle east	2	110/156	0.72	0.31 to 0.98	96%	<0.01
**Types of vaccine**						
Moderna only	2	50/71	0.58	0.12 to 0.97	91%	<0.01
Pfizer only	4	505/731	0.67	0.48 to 0.83	95%	<0.01
mRNA vaccines + vector vaccines	1	116/138	0.84	0.77 to 0.90	NA	NA
**Publish year**						
2021	3	121/151	0.72	0.45 to 0.92	89%	<0.01
2022	4	550/789	0.65	0.48 to 0.81	95%	<0.01

* CI: confidence interval; M-H: Mantel-Haenszel; No.: number; RR: risk ratio.

## Abbreviations

OLT: orthotopic liver transplant
SARS-CoV-2: severe acute respiratory syndrome coronavirus 2
ORs: odds ratios
COVID-19: Coronavirus disease 2019
PROSPERO: International Prospective Register of Systematic Reviews
MOOSE: Meta-analysis of Observational Studies in Epidemiology
PRISMA: Preferred Reporting Items for Systematic Reviews and Meta-Analyses
PICO: Patient/Problem, Intervention, Comparison, and Outcome
MeSH: Medical Subject Headings
RoBANS: Risk of Bias Assessment tool for Nonrandomised Studies
CKD: chronic kidney disease
