# Supplementary material for: Humoral Immunogenicity to SARS-CoV-2 Vaccination in Liver Transplant Recipients: A Systematic Review and Meta-Analysis
Source: Int J Biol Sci. 2022 Sep 21;18(15):5849–57. doi: 10.7150/ijbs.77030 (PMC9576515; doi:10.7150/ijbs.77030)
Supplement: Supplementary file 1 — Supplementary materials and methods, figures and tables. [file ijbsv18p5849s1.pdf]

Supplementary Material

Humoral immune response to severe acute respiratory syndrome coronavirus-2 vaccination in liver transplant recipients

Table of Contents

I. Supplementary Materials and Methods……………………………………..…..….2

Search strategy and results

Risk of bias

II. Supplementary Tables.……………………………………………….……….….8

III. Supplementary Figures....……………..…………………………….………..….11

I. Supplementary Materials and Methods

1. Search strategy and results

(1) Summary of search results

No.	DB	Results	Duplication
1	PubMed (Medline)	66	90
2	EMBASE	267	
4	Cochrane Library	19	
Number of Search Results (including duplication)		352	
**Number of Search Results (without duplication)**		**262**	

(2) Keywords

PICO	Fields	Keywords	Remarks
P	MeSH	Liver transplantation	A
	TIAB	Liver transplant*	B
	TIAB	Liver grafting*	
	TIAB	Hepatic transplant*	
		**A OR B**	C
I	MeSH	COVID-19 Vaccines	D
	MeSH	SARS-CoV-2	
	MeSH	Covid-19	
	MeSH	Coronavirus	
	MeSH	Ad26COVS1	
	MeSH	2019-nCoV Vaccine mRNA-1273	
	MeSH	BNT162 vaccine	
	MeSH	ChAdOx1 nCoV-19	
	TIAB	COVID-19 vaccin*	E
	TIAB	COVID-19 virus vaccin*	
	TIAB	COVID19 virus vaccin*	
	TIAB	COVID19 vaccin*	
	TIAB	COVID-19 pandemic	
	TIAB	Coronavirus disease-19	

	TIAB	SARS-CoV-2	
	TIAB	SARS2 vaccin*	
	TIAB	Coronavirus disease 2019	
	TIAB	Coronavirus disease 19	
	TIAB	2019-nCoV vaccin*	
	TIAB	SARS-CoV-2 vaccin*	
	TIAB	SARS coronavirus 2 vaccin*	
	TIAB	2019 novel coronavir*	
	TIAB	2019-nCoV	
	TIAB	Wuhan coronavir*	
	TIAB	Severe acute respiratory syndrome coronavirus 2	
	TIAB	Janssen	
	TIAB	JNJ-78436735	
	TIAB	JNJ78436735	
	TIAB	Johnson and Johnson Covid-19 vaccin*	
	TIAB	COVID-19 vaccine Johnson and Johnson	
	TIAB	Moderna	
	TIAB	2019 nCoV Vaccine mRNA 1273	
	TIAB	Moderna COVID-19 vaccin*	
	TIAB	Elasomeran	
	TIAB	Moderna COVID-19 vaccine RNA	
	TIAB	COVID-19 vaccine moderna	
	TIAB	mRNA-1273	
	TIAB	COVID-19 Vaccine Pfizer-BioNTech	
	TIAB	BNT 162 vaccin*	
	TIAB	BNT162 vaccin*	
	TIAB	Pfizer	
	TIAB	Pidacmeran	
	TIAB	Abdavomeran	
	TIAB	Tozinameran	
	TIAB	Comirnaty	
	TIAB	AstraZeneca	
	TIAB	ChAdOx1 nCoV-19	
	TIAB	Oxford-AstraZeneca COVID-19 vaccin*	
	TIAB	ChAdOx1 COVID 19 vaccine*	

	TIAB	Covishield	
	TIAB	Vaxzevria	
	TIAB	AZD1222	
	TIAB	AZD-1222	
	TIAB	Coronavir*	
	TIAB	mRNA vaccin*	
	TIAB	ad26.cov2.s vaccine	
	TIAB	Ad26COVS1	
		**D OR E**	**F**
	MeSH	Immunogenicity, vaccine	
	MeSH	Antibodies	
	MeSH	Antibodies, viral	
	MeSH	Antibody formation	
	TIAB	Seroprevalence*	
	TIAB	Immunogenicit*	
	TIAB	Vaccine antigenicit*	
	TIAB	Antibod*	
	TIAB	Anti-bod*	**H**
	TIAB	Viral antibod*	
	TIAB	Antibody formation*	
	TIAB	Antibody response*	
	TIAB	Antibody production*	
		**G OR H**	**I**
		**C AND F AND I**	

(3) Search strategy

DB	Search Strategy

**PubMed**	("liver transplantation"[MeSH Terms] OR ("liver transplant*"[Title/Abstract] OR "liver grafting*"[Title/Abstract] OR "hepatic transplant*"[Title/Abstract])) AND ("covid 19 vaccines"[MeSH Terms] OR "SARS-CoV-2"[MeSH Terms] OR "covid 19"[MeSH Terms] OR "coronavirus"[MeSH Terms] OR "Ad26COVS1"[MeSH Terms] OR "2019 nCoV Vaccine mRNA 1273"[MeSH Terms] OR "bnt162 vaccine"[MeSH Terms] OR "ChAdOx1 nCoV-19"[MeSH Terms] OR ("covid 19 vaccin*"[Title/Abstract] OR "covid 19 virus vaccin*"[Title/Abstract] OR "covid19 vaccin*"[Title/Abstract] OR "COVID-19 pandemic"[Title/Abstract] OR "coronavirus disease 19"[Title/Abstract] OR "SARS-CoV-2"[Title/Abstract] OR "Coronavirus disease 2019"[Title/Abstract] OR "coronavirus disease 19"[Title/Abstract] OR "2019 ncov vaccin*"[Title/Abstract] OR "sars cov 2 vaccin*"[Title/Abstract] OR "sars coronavirus 2 vaccin*"[Title/Abstract] OR "2019 novel coronavir*"[Title/Abstract] OR "2019-nCoV"[Title/Abstract] OR "wuhan coronavir*"[Title/Abstract] OR "Severe acute respiratory syndrome coronavirus 2"[Title/Abstract] OR "Janssen"[Title/Abstract] OR "JNJ-78436735"[Title/Abstract] OR "JNJ78436735"[Title/Abstract] OR "johnson and johnson covid 19 vaccin*"[Title/Abstract] OR "Moderna"[Title/Abstract] OR "2019 nCoV Vaccine mRNA 1273"[Title/Abstract] OR "moderna covid 19 vaccin*"[Title/Abstract] OR "Elasomeran"[Title/Abstract] OR "COVID-19 vaccine moderna"[Title/Abstract] OR "mRNA-1273"[Title/Abstract] OR "COVID-19 vaccine Pfizer-BioNTech"[Title/Abstract] OR "bnt162 vaccin*"[Title/Abstract] OR "Pfizer"[Title/Abstract] OR "Tozinameran"[Title/Abstract] OR "Comirnaty"[Title/Abstract] OR "AstraZeneca"[Title/Abstract] OR "ChAdOx1 nCoV-19"[Title/Abstract] OR "oxford astrazeneca covid 19 vaccin*"[Title/Abstract] OR "chadox1 covid 19 vaccine*"[Title/Abstract] OR "Covishield"[Title/Abstract] OR "Vaxzevria"[Title/Abstract] OR "AZD1222"[Title/Abstract] OR "AZD-1222"[Title/Abstract] OR "coronavir*"[Title/Abstract] OR "mrna vaccin*"[Title/Abstract] OR "ad26.cov2.s vaccine"[Title/Abstract] OR "Ad26COVS1"[Title/Abstract])) AND ("immunogenicity, vaccine"[MeSH Terms] OR "antibodies"[MeSH Terms] OR "antibodies, viral"[MeSH Terms] OR "antibody formation"[MeSH Terms] OR ("seroprevalence*"[Title/Abstract] OR "immunogenicit*"[Title/Abstract] OR "vaccine antigenicit*"[Title/Abstract] OR "antibod*"[Title/Abstract] OR "anti bod*"[Title/Abstract] OR "viral antibod*"[Title/Abstract] OR "antibody formation*"[Title/Abstract] OR "antibody response*"[Title/Abstract] OR "antibody production*"[Title/Abstract]))
**EMBASE**	(('liver transplantation'/exp OR 'liver graft'/exp) OR ('liver transplant*':ab,ti OR 'liver graft':ab,ti OR 'hepatic transplant*':ab,ti)) AND (('sars-cov-2 vaccine'/exp OR 'severe acute respiratory syndrome coronavirus 2'/exp OR 'coronavirus disease 2019'/exp OR 'coronavirinae'/exp OR 'ad26.cov2.s vaccine'/exp OR 'elasomeran'/exp OR 'bnt 162 vaccine'/exp OR 'vaxzevria'/exp) OR ('covid-19 vaccin*':ab,ti OR 'covid-19 virus vaccin*':ab,ti OR 'covid19 virus vaccin*':ab,ti OR 'covid19 vaccin*':ab,ti OR 'covid-19 pandemic':ab,ti OR 'coronavirus disease-19':ab,ti OR 'sars-cov-2':ab,ti OR 'sars2 vaccin*':ab,ti OR 'coronavirus disease 2019':ab,ti OR 'coronavirus disease 19':ab,ti OR '2019-ncov vaccin*':ab,ti OR 'sars-cov-2 vaccin*':ab,ti OR 'sars coronavirus 2 vaccin*':ab,ti OR '2019 novel coronavir*':ab,ti OR '2019-ncov':ab,ti OR 'wuhan coronavir*':ab,ti OR 'severe acute respiratory syndrome coronavirus 2':ab,ti OR 'janssen':ab,ti OR 'jnj-78436735':ab,ti OR 'jnj78436735':ab,ti OR 'johnson and johnson covid-19 vaccin*':ab,ti OR 'covid-19 vaccine johnson and johnson':ab,ti OR 'moderna':ab,ti OR '2019 ncov vaccine mrna 1273':ab,ti OR 'moderna covid-19 vaccin*':ab,ti OR 'elasomeran':ab,ti OR 'moderna covid-19 vaccine rna':ab,ti OR 'covid-19 vaccine moderna':ab,ti OR 'mrna-1273':ab,ti OR 'covid-19 vaccine pfizer-biontech':ab,ti OR 'bnt 162 vaccin*':ab,ti OR 'bnt162 vaccin*':ab,ti OR 'pfizer':ab,ti OR 'pidacmeran':ab,ti OR 'abdavomeran':ab,ti OR 'tozinameran':ab,ti OR 'comirnaty':ab,ti OR 'astrazeneca':ab,ti OR 'chadox1 ncov-19':ab,ti OR 'oxford-astrazeneca covid-19 vaccin*':ab,ti OR 'chadox1 covid 19 vaccine*':ab,ti OR 'covishield':ab,ti OR 'vaxzevria':ab,ti OR 'azd1222':ab,ti OR 'azd-1222':ab,ti OR 'coronavir*':ab,ti OR 'mrna vaccin*':ab,ti OR 'ad26.cov2.s vaccine':ab,ti OR 'ad26covs1':ab,ti)) AND (('vaccine immunogenicity'/exp OR 'antibody'/exp OR 'virus antibody'/exp OR 'antibody production'/exp OR 'seroprevalence'/exp OR 'immunogenicity'/exp OR (antibodies,antisera AND 'immunoglobulins'/exp)) OR ('seroprevalence*':ab,ti OR 'immunogenicit*':ab,ti OR 'vaccine antigenicit*':ab,ti OR 'antibod*':ab,ti OR 'anti-bod*':ab,ti OR 'viral antibod*':ab,ti OR 'antibody formation*':ab,ti OR 'antibody response*':ab,ti OR 'antibody production*':ab,ti))

**Cochrane**	((MeSH descriptor: [Liver Transplantation] explode all trees) OR (Liver Transplant*):ti,ab,kw OR (Liver Grafting*):ti,ab,kw OR (Hepatic Transplant*):ti,ab,kw) AND ((MeSH descriptor: [COVID-19 Vaccines] explode all trees OR MeSH descriptor: [SARS-CoV-2] explode all trees OR MeSH descriptor: [COVID-19] explode all trees OR MeSH descriptor: [Coronavirus] explode all trees OR MeSH descriptor: [Ad26COVS1] explode all trees OR MeSH descriptor: [2019-nCoV Vaccine mRNA-1273] explode all trees OR MeSH descriptor: [BNT162 Vaccine] explode all trees OR MeSH descriptor: [ChAdOx1 nCoV-19] explode all trees) OR ((COVID-19 vaccin*):ti,ab,kw OR (COVID-19 virus vaccin*):ti,ab,kw OR (COVID19 virus vaccin*):ti,ab,kw OR (COVID19 vaccin*):ti,ab,kw OR (COVID-19 pandemic):ti,ab,kw OR (Coronavirus disease-19):ti,ab,kw OR (SARS-CoV-2):ti,ab,kw OR (SARS2 vaccin*):ti,ab,kw OR (Coronavirus disease 2019 vaccin*):ti,ab,kw OR (Coronavirus disease 19 vaccin*):ti,ab,kw OR (2019 nCoV vaccin*):ti,ab,kw OR (SARS-CoV-2 vaccin*):ti,ab,kw OR (SARS coronavirus 2 vaccin*):ti,ab,kw OR (2019 novel coronavirus*):ti,ab,kw OR (2019 nCoV):ti,ab,kw OR (Wuhan coronavirus*):ti,ab,kw OR (severe acute respiratory syndrome coronavirus 2):ti,ab,kw OR (Janssen):ti,ab,kw OR (JNJ-78436735):ti,ab,kw OR (JNJ78436735):ti,ab,kw OR (Johnson and Johnson Covid-19 vaccin*):ti,ab,kw OR (COVID-19 vaccine Johnson and Johnson):ti,ab,kw OR (Moderna):ti,ab,kw OR (2019 nCoV Vaccine mRNA 1273):ti,ab,kw OR (Moderna COVID-19 vaccin*):ti,ab,kw OR (Elasomeran):ti,ab,kw OR (Moderna COVID-19 vaccine RNA):ti,ab,kw OR (COVID-19 vaccine moderna):ti,ab,kw OR (mRNA-1273):ti,ab,kw OR (COVID-19 Vaccine Pfizer-BioNTech):ti,ab,kw OR (BNT 162 vaccin*):ti,ab,kw OR (BNT162 vaccin*):ti,ab,kw OR (Pfizer):ti,ab,kw OR (Pidacmeran):ti,ab,kw OR (Abdavomeran):ti,ab,kw OR (Tozinameran):ti,ab,kw OR (Comirnaty):ti,ab,kw OR (AstraZeneca):ti,ab,kw OR (ChAdOx1 nCoV-19):ti,ab,kw OR (Oxford-AstraZeneca COVID-19 vaccin*):ti,ab,kw OR (ChAdOx1 COVID 19 vaccine*):ti,ab,kw OR (Covishield):ti,ab,kw OR (Vaxzevria):ti,ab,kw OR (AZD1222):ti,ab,kw OR (AZD-1222):ti,ab,kw OR (Coronavir*):ti,ab,kw OR (mRNA vaccin*):ti,ab,kw OR (ad26.cov2.s vaccine):ti,ab,kw OR (Ad26COVS1):ti,ab,kw)) AND ((MeSH descriptor: [Immunogenicity, Vaccine] explode all trees OR MeSH descriptor: [Antibodies] explode all trees OR MeSH descriptor: [Antibodies, Viral] explode all trees OR MeSH descriptor: [Antibody Formation] explode all trees) OR ((Seroprevalence*):ti,ab,kw OR (Immunogenicit*):ti,ab,kw OR (Vaccine antigenicit*):ti,ab,kw OR (Antibod*):ti,ab,kw OR (Anti-bod*):ti,ab,kw OR (Viral antibod*):ti,ab,kw OR (Antibody formation*):ti,ab,kw OR (Antibody response*):ti,ab,kw OR (Antibody production*):ti,ab,kw)) NOT (MeSH descriptor: [Animals] this term only NOT (MeSH descriptor: [Animals] this term only AND MeSH descriptor: [Humans] this term only))

2. Risk of bias

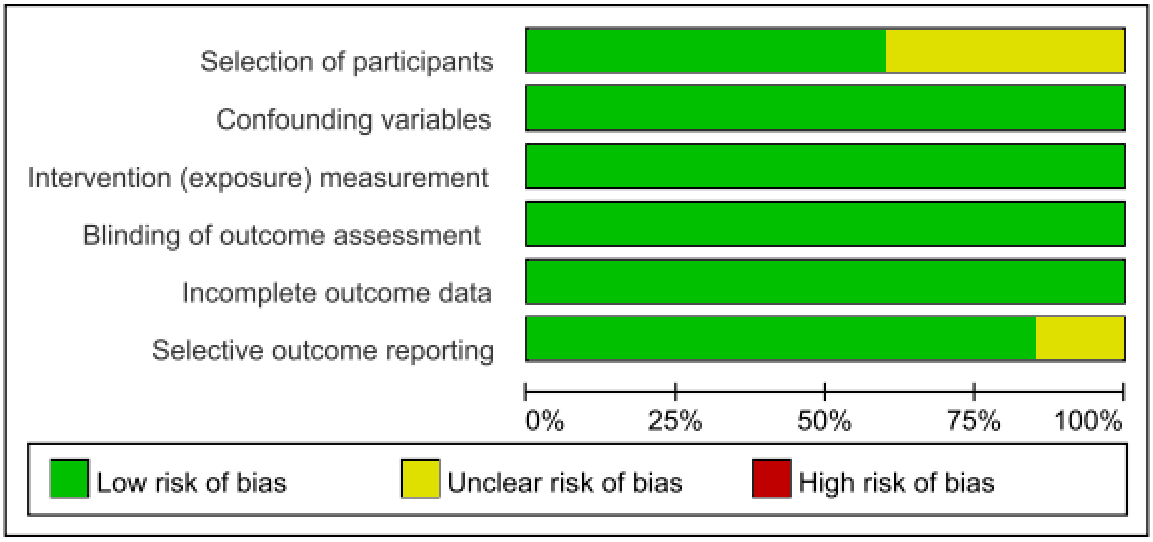

	Selection of participants	Confounding variables	Intervention (exposure) measurement	Blinding of outcome assessment	Incomplete outcome data	Selective outcome reporting
Cholankeril 2021	+	+	+	+	+	+
Davidov 2022	+	+	+	+	+	+
D'Offizi 2021	?	+	+	+	+	+
Erol 2021	+	+	+	+	+	+
Fernández-Ruiz 2021	+	+	+	+	+	+
Guarino 2022	+	+	+	+	+	+
Herrera 2021	+	+	+	+	+	+
Huang 2022	?	+	+	+	+	?
Marion 2022	?	+	+	+	+	?
Mulder 2022	?	+	+	+	+	+
Nazaruk 2021	?	+	+	+	+	+
Rabinowich 2021	+	+	+	+	+	+
Rahav 2021	+	+	+	+	+	?
Rashidi-Alavijeh 2021	+	+	+	+	+	+
Ruether 2022	+	+	+	+	+	+
Sakai 2022	?	+	+	+	+	+
Strauss 2021	+	+	+	+	+	+
Thuluvath 2021	+	+	+	+	+	+
Timmermann 2021	?	+	+	+	+	+
Toniutto 2022	?	+	+	+	+	+

II. Supplementary Tables

Supplementary Table 1. Summary of the immunogenicity rates of COVID-vaccination in patients with liver transplantation recipients vs. control groups

Subgroup/Subset	No. of studies	No. of patients, responder/total (LT recipients)	No. of patients, responder/total (controls)	OR (M-H, Random)	95% CI	I^2	P for heterogeneity
**LT recipients vs. control, overall**	16	1049/1592	1318/1602	**0.80**	**0.69 to 0.92**	93%	<0.01
**LT recipients vs. healthy control**	10	750/1078	1008/1029	**0.69**	**0.63 to 0.77**	81%	<0.01
**LT recipients vs. KT recipients**	6	256/443	335/867	**1.50**	**1.35 to 1.67**	0%	1.00
LT recipients vs. HT recipients	3	219/368	108/226	1.44	0.89 to 2.32	92%	<0.01
**LT recipients vs. liver cirrhosis**	2	100/200	109/127	**0.54**	**0.47 to 0.62**	0%	0.61

* CI: confidence interval; M-H: Mantel-Haenszel; No.: number; OR; odds ratio; KT, kidney transplantation; HT, heart transplantation

Supplementary Table 2. Meta-regression for the immunogenicity rates of COVID-vaccination in patients with liver transplantation recipients

Variable	Coefficient (95% CI)	p-value
Age (year)	-0.001 (-0.023, 0.021)	0.946
Male (%)	0.003 (-0.002, 0.010)	0.184
BMI (kg/m²)	-0.067 (-1.349, 0.030)	0.177
Obesity (BMI >30) (%)	-0.005 (-0.020, 0.009)	0.491
Diabetes (%)	0.001 (-0.012, 0.015)	0.197
Hypertension (%)	-0.011 (-0.023, 0.001)	0.062
Chronic kidney disease (%)	-0.006 (-0.012, 0.005)	0.052
Time from LT to vaccination (year)	0.009 (-0.007, 0.026)	0.274
Use of calcineurin inhibitor (%)	-0.002 (-0.006, 0.001)	0.286
Use of MMF (%)	-0.005 (-0.012, 0.002)	0.171
Use of prednisolone (%)	-0.002 (-0.010, 0.004)	0.459
Use of mTOR inhibitor (%)	-0.001 (-0.006, 0.005)	0.846
**Immunosuppressant (1 agent) (%)**	**0.005 (0.001, 0.008)**	**0.002**
Immunosuppressant (2 agent) (%)	-0.003 (-0.007, 0.002)	0.239
Immunosuppressant (3 agent) (%)	-0.005 (-0.019, 0.008)	0.469

Supplementary Table 3. Risk factors for the unresponsiveness to vaccination in liver transplantation recipients

Potential risk factors	No. of studies	Pooled odds ratio	95% CI	I^2	P for heterogeneity
**Male gender**	3	**4.40**	**1.54 to 12.58**	0%	0.49
**Old age**	6	**2.82**	**2.10 to 3.80**	0%	0.92
**Chronic kidney disease**	4	**27.56**	**10.06 to 87.54**	95%	<0.01
**Obesity**	3	**2.64**	**1.14 to 6.09**	17%	0.30
**Multi-immunosuppressants**	6	**10.40**	**6.12 to 17.68**	91%	<0.01
**High dose steroid**	2	**5.41**	**1.66 to 17.67**	0%	0.74
**High dose MMF**	5	**5.22**	**3.66 to 7.45**	0%	0.80
High dose tacrolimus	2	2.90	0.92 to 9.09	0%	0.84
**Time since liver transplantation**	2	**4.51**	**1.46 to 13.97**	64%	0.09
**Vaccination in 1st year of transplantation**	3	**18.53**	**7.67 to 44.79**	91%	<0.01

III. Supplementary Figures

Supplementary Figure 1. Funnel plots of immunogenicity rates

(A) Pooled immunogenicity rate, (B) Comparison of liver transplantation recipients and control groups

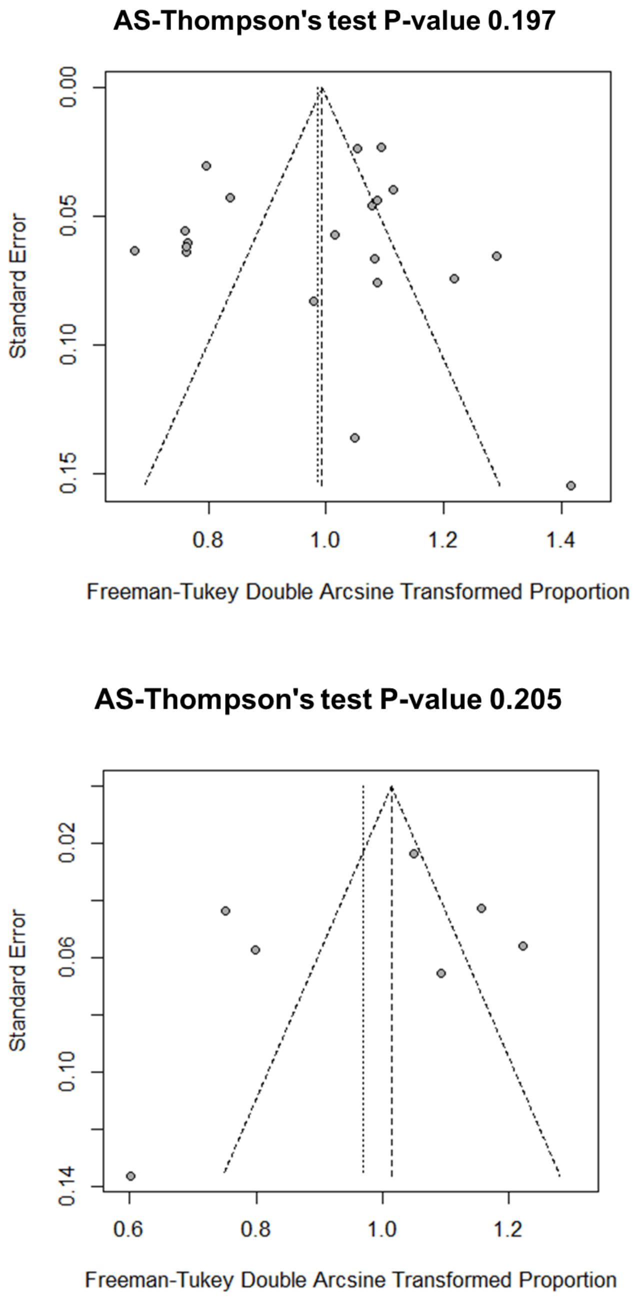

Supplementary Figure 2. Funnel plots of any type of adverse events after the COVID-19 vaccine

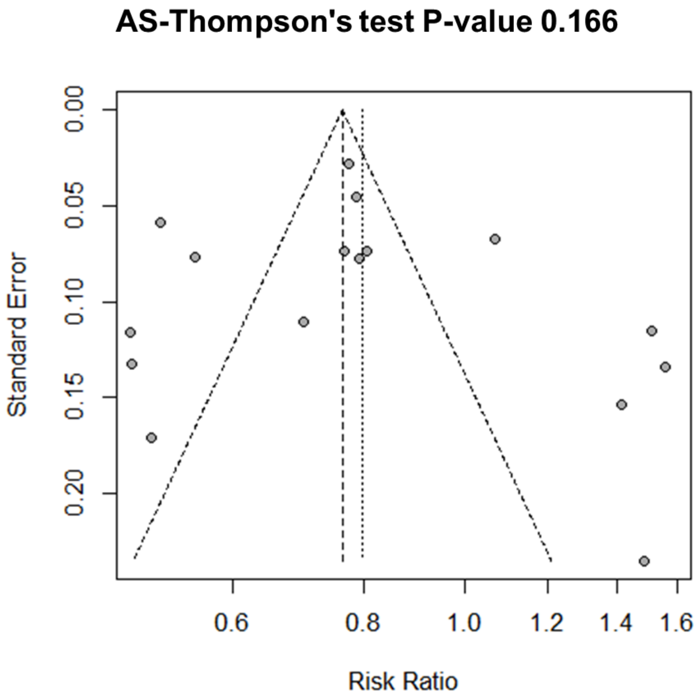

Supplementary Figure 3. Flowchart of the study inclusion and exclusion process of the systematic review

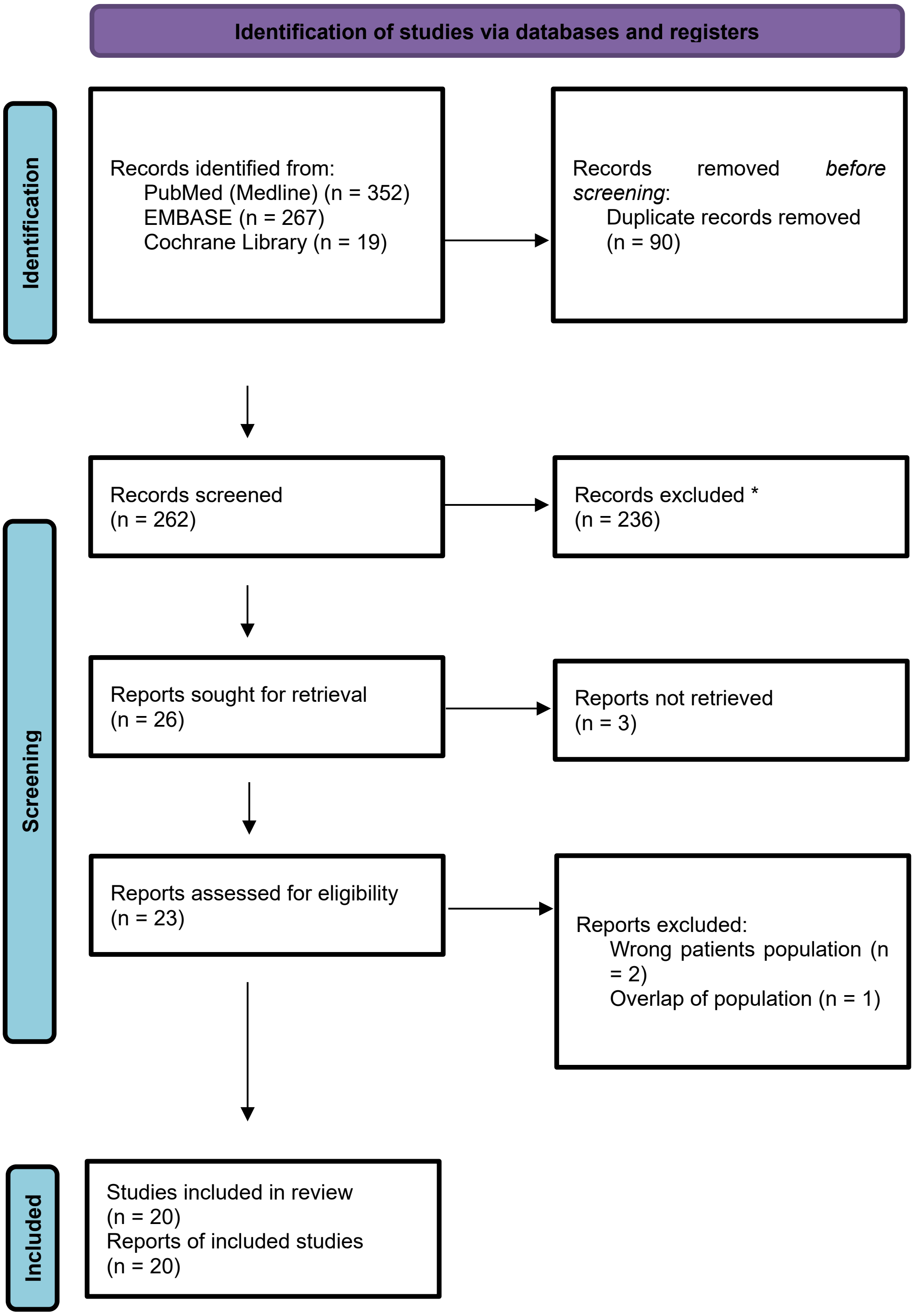

*irrelevant subjects, systematic reviews, case report, abstract only, non-English material

From: Page MJ, McKenzie JE, Bossuyt PM, Boutron I, Hoffmann TC, Mulrow CD, et al. The PRISMA 2020 statement: an updated guideline for reporting systematic reviews. BMJ 2021;372:n71. doi: 10.1136/bmj.n71
